# Application of computerized 3D-CT texture analysis of pancreas for the assessment of patients with diabetes

**DOI:** 10.1371/journal.pone.0227492

**Published:** 2020-01-13

**Authors:** Siwon Jang, Jung Hoon Kim, Seo-Youn Choi, Sang Joon Park, Joon Koo Han

**Affiliations:** 1 Department of Radiology, SMG—SNU Boramae Medical Center, Seoul, Korea; 2 Department of Radiology, Seoul National University Hospital, Seoul, Korea; 3 Institute of Radiation Medicine, Seoul National University College of Medicine, Seoul, Korea; 4 Department of Radiology, Soonchunhyang University College of Medicine, Soonchunhyang University Bucheon Hospital, Bucheon, Korea; Shanghai Diabetes Institute, CHINA

## Abstract

**Objective:**

To evaluate the role of computerized 3D CT texture analysis of the pancreas as quantitative parameters for assessing diabetes.

**Methods:**

Among 2,493 patients with diabetes, 39 with type 2 diabetes (T2D) and 12 with type 1 diabetes (T1D) who underwent CT using two selected CT scanners, were enrolled. We compared these patients with age-, body mass index- (BMI), and CT scanner-matched normal subjects. Computerized texture analysis for entire pancreas was performed by extracting 17 variable features. A multivariate logistic regression analysis was performed to identify the predictive factors for diabetes. A receiver operator characteristic (ROC) curve was constructed to determine the optimal cut off values for statistically significant variables.

**Results:**

In diabetes, mean attenuation, standard deviation, variance, entropy, homogeneity, surface area, sphericity, discrete compactness, gray-level co-occurrence matrix (GLCM) contrast, and GLCM entropy showed significant differences (*P* < .05). Multivariate analysis revealed that a higher variance (adjusted OR, 1.002; *P* = .005), sphericity (adjusted OR, 1.649×10^4^; *P* = .048), GLCM entropy (adjusted OR, 1.057×10^5^; *P* = .032), and lower GLCM contrast (adjusted OR, 0.997; *P* < .001) were significant variables. The mean AUCs for each feature were 0.654, 0.689, 0.620, and 0.613, respectively (*P* < .05). In subgroup analysis, only larger surface area (adjusted OR, 1.000; *P* = .025) was a significant predictor for T2D.

**Conclusions:**

Computerized 3D CT texture analysis of the pancreas could be helpful for predicting diabetes. A higher variance, sphericity, GLCM entropy, and a lower GLCM contrast were the significant predictors for diabetes.

## Introduction

Diabetes, a lifelong condition that causes glucose dysmetabolism, is one of the most prevalent chronic metabolic diseases worldwide. It is a major cause of blindness, kidney failure, heart attacks, stroke, and lower limb amputation. The early diagnosis and treatment of diabetes reduce not only the risk of occurrence and progression of short-term microvascular complications [1, 2], but also long-term, major microvascular complications and mortality [3]. As the global prevalence of diabetes is increasing, it has become more important to stimulate the adoption of effective measures for the surveillance, prevention, and control of diabetes and its complications. Many countries are adopting surveillance programs for the high risk population, however, up to this point, the effort is confined to laboratory testing using plasma glucose level or hemoglobin A1c (HbA1c) [4].

Recent studies have shown that both type 1 diabetes (T1D) and type 2 diabetes (T2D) are characterized by a deficit in ß-cells, a pancreatic endocrine cell that secretes insulin [5]. A diabetic pancreas may also present with fibrosis and atrophy due to glandular replacement by connective tissue and round cells [5], which may alter the texture parameters of the pancreatic parenchyma. Therefore, if we could trace pathological changes in the pancreas, it would be helpful in order to obtain a better understanding of the natural history of diabetes. Indeed, there have been various approaches for evaluating the in vivo pancreatic endocrine function using CT [6–9] as the use of CT for diagnosis and follow-up of diseases affecting abdominal organs has dramatically increased over the past several decades.

Texture analysis is a quantitative imaging analysis tool that uses attenuation values of each voxel and their distribution within target lesions, and is expected to allow a more detailed information using quantitative assessment of lesion characteristics than visual analysis by human observers [10]. It has been used in the field of both oncologic and nononcologic imaging in order to predict pathologic features, response to therapy, and prognosis [11–14]. For example, whole-liver CT-texture analysis was proven to have potential to predict patients at risk of developing early liver metastases in colorectal cancer [15]. As CT texture features reflect information regarding the tissue microenvironment, they can be used in predicting the development of disease before a lesion actually becomes visible. Given that CT texture features may reflect histologic changes in diabetic pancreas, monitoring changes in these features could potentially alert radiologists and clinicians to the imminent development or progression of diabetes. In this context, computerized texture analysis of the pancreas can be a useful tool for the surveillance of diabetes, especially for the patients who had undergone CT scans for various purposes, without being tested for diabetes. However, no study has yet performed texture analysis of the pancreatic parenchyma using CT. For this purpose, we performed computerized CT texture analysis on pancreatic parenchyma for the quantitative assessment in patients having different type and duration of diabetes, and in age- and BMI-matched normal subjects.

## Materials and methods

### Study population

**Fig 1:**
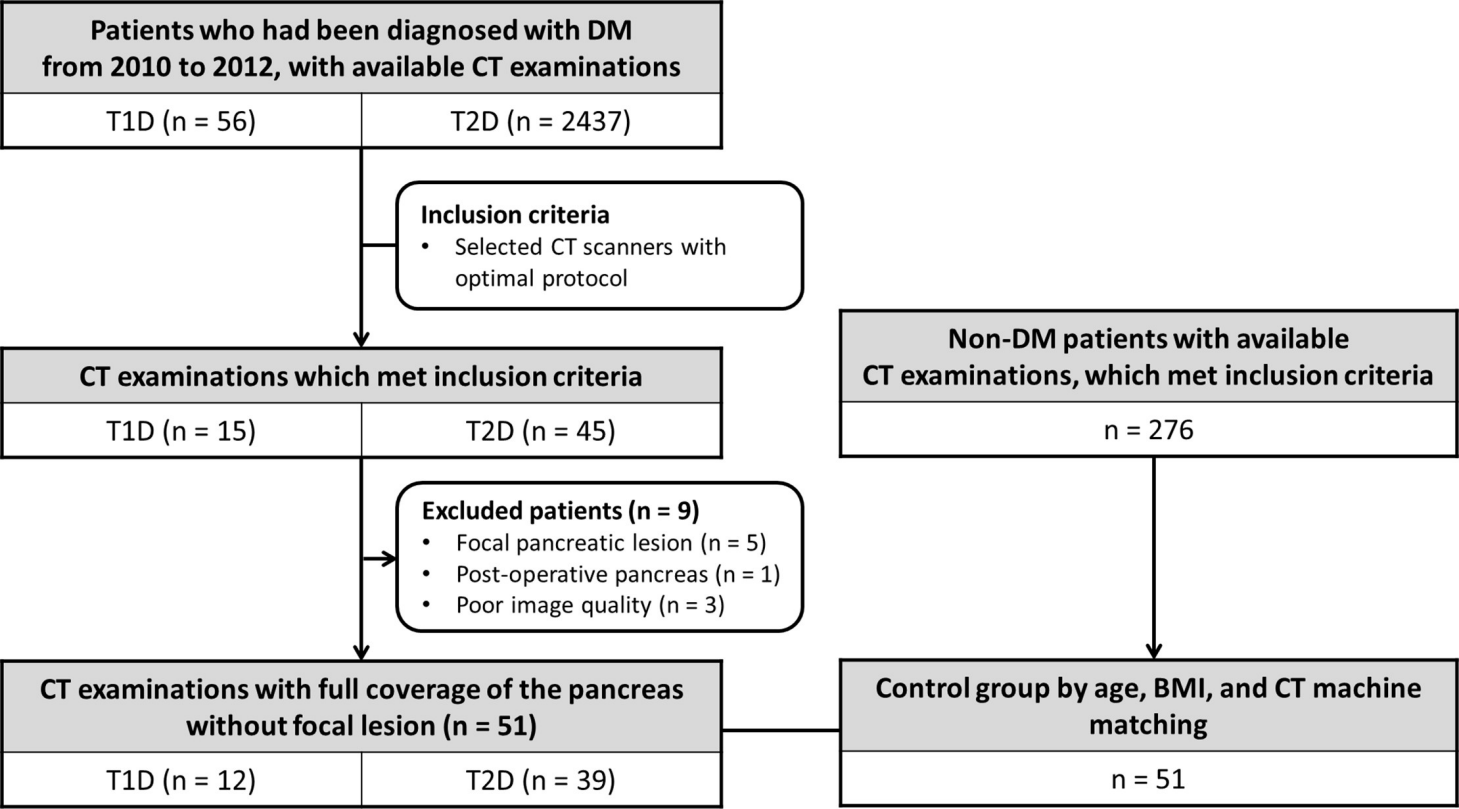
Flow chart of study population. The flowchart shows how the study population and the control group were selected.

This retrospective study was approved by our institutional review board in Seoul National University Hospital (IRB No. 1805-100-946), and informed consent was waived. All patient data was anonymized before analysis by the authors. We searched the electronic medical records and the hospital information systems from 2010 to 2012, and selected patients who had been diagnosed with either T1D or T2D, and who also had available CT examinations. “Insulin-dependent diabetes mellitus” and “Juvenile diabetes mellitus” were regarded as T1D, whereas “Non-Insulin-dependent diabetes mellitus” and “diabetes mellitus” were regarded as T2D. A total of 56 T1D patients and 2,437 T2D patients were searched. We then selected patients with the following inclusion criteria: 1) CT scans using two selected CT scanners including a Sensation 16 (16-channel scanner, Siemens Medical Solutions) or a Brilliance 64 (64-channel scanner, Philips Healthcare); 2) Examination protocols which included venous phase images with a section thickness of 5 mm or less. After selecting patients with appropriate CT images, we further excluded those who had a focal pancreatic lesion (n = 5), who had undergone pancreatic surgery (n = 1), and whose images had severe artifact (n = 3). Finally, our study population consisted of 12 T1D patients (mean age, 48.7 ± 12.8; age range, 33–69 years) and 39 T2D patients (mean age, 57.3 ± 6.4; age range, 44–69 years). The T2D group was again divided into two subgroups according to the patients’ insulin dependence, i.e. under insulin therapy (n = 11) vs. under oral anti-diabetic treatment (n = 28). For the control group, we searched the electronic medical records and the hospital information systems by matching the CT unit, patient age, and BMI. Our final control groups consisted of 51 corresponding patients (mean age, 55.3 ± 9.1) according to the CT unit, patient age, and BMI matching (a range of ±2 years in age and ±0.5 kg/m^2^ of BMI). Fig 1 shows the flowchart of this study population. The patient BMI, serum HbA1c level, and casual blood glucose level were obtained from the patient’s medical records. Plasma glucose levels were measured using Hitachi 747 chemistry analyzer (Hitachi, Tokyo, Japan).

### CT examination

CT examinations were performed using the following two CT scanners: Sensation 16 (n = 74), and Brilliance 64 (n = 28). For 16- and 64-detector CT examinations, detector collimations of 0.75 and 0.625 mm, respectively, were used. A section thickness of 2.5–5.0 mm with a 2- to 3- mm reconstruction interval, a field of view of 300–370 mm, a gantry rotation time of 0.5 s, an effective amperage setting of 150–200 mAs, and a peak voltage of 120 kVp were used for all of the CT scanners. All of the patients’ CT protocols included venous phase images. For dynamic phase imaging, a fixed dose of 1.5–2.0 mL of nonionic contrast material (iopromide [370 mg of iodine per millimeter], Ultravist 370; Bayer HealthCare) per kilogram of body weight (555 mgI/kg) was injected at a rate of 2.0–4.0 mL/sec using a power injector (Multilevel CT; Medrad). The venous phase scans were obtained 70–80 seconds after administration of the IV contrast material.

### Computerized texture analysis

**Fig 2:**
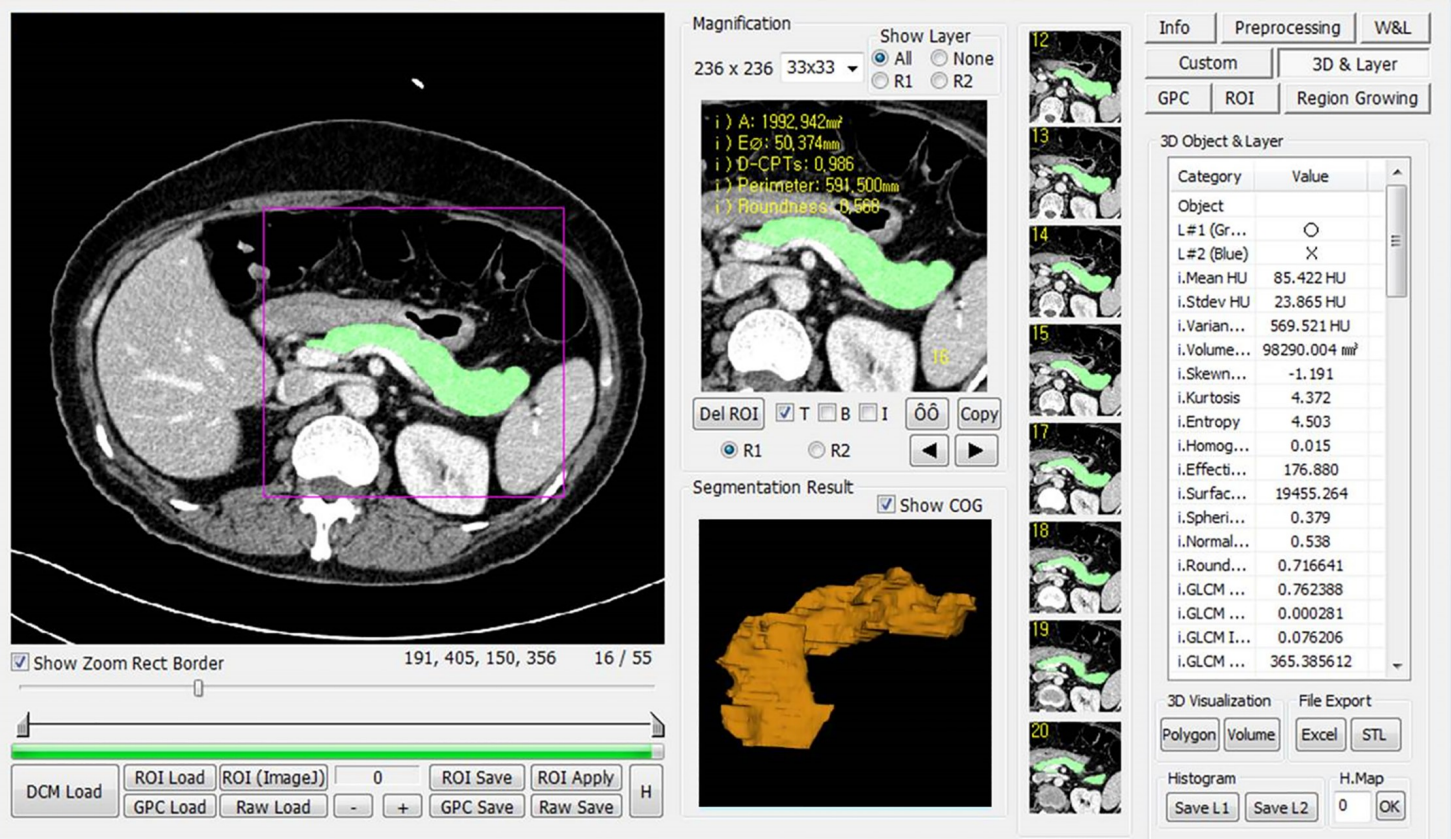
The screenshot shows the texture analysis software program. The segmentation of pancreatic parenchyma was manually conducted using an in-house software program, and texture features of the pancreatic parenchyma were automatically extracted and calculated by the software program.

The in-house developed software program (MISSTA; Medical Imaging Solution for Segmentation and Texture Analysis), which was coded in the C++ language with MFC (Microsoft Foundation Classes, Microsoft, Redmond, WA), was used for automated quantification of the morphologic and textural parameters of the pancreatic parenchyma. It adopted a statistical-based model to describe the relationship of the gray-level values in the image. Pancreatic areas were selected as regions of interest (ROI) that contained the pancreatic parenchyma, which were manually drawn in each slice of the venous phase images by a radiologist (S.J., with three years of experience in abdominal radiology) and were confirmed by another radiologist (J.H.K., with 17 years of experience in abdomen CT). It automatically calculated the texture and first order features using the input ROI information. The texture analysis process is presented in Fig 2. The histogram parameters analyzed included mean attenuation, standard deviation, skewness, kurtosis, entropy, and homogeneity. The volumetric parameters included surface area, effective diameter, volume, and sphericity. Finally, the following texture parameters were obtained from the pancreatic parenchyma: discrete compactness; gray-level co-occurrence matrix (GLCM) contrast; GLCM entropy; GLCM angular second moment (ASM); GLCM inverse difference moment (IDM); and GLCM moments. See the S1 Appendix for detailed information regarding the texture features.

### Statistical analysis

All data were analyzed using SPSS version 21.0 (IBM Corporation) and MedCalc software (version 12.6.1.0 for Microsoft Windows 2000/XP/Vista/7; MedCalc Software). *P* values less than .05 were considered to be significant. To compare the variables between diabetic patients and the control group, the independent sample *t* test was used. Thereafter, logistic regression analysis using the backward elimination method with texture parameters shown to be of statistical significance in univariate analysis, was performed to identify significant independent predictors for diabetes. The receiver operator characteristic (ROC) curve was constructed to determine the optimal cut-off values for statistically significant variables. Subgroup analyses were also performed in the same manner as total group analyses. In addition to a comparison between diabetic patients and the control group, we also performed the Mann-Whitney U test to compare the T1D and T2D groups.

## Results

The characteristics of the study populations are shown in Table 1. The mean age was significantly higher in T2D patients (57.3 ± 6.4 vs. 48.7 ± 12.8, *P* = 0.043). Alternatively, the mean disease duration was significantly shorter in T2D patients (5.2 ± 6.2 vs. 15.9 ± 11.6, *P* = 0.009). The HbA1c level also showed a significant difference between two groups, i.e. being lower in the T2D groups (7.4 ± 1.4 vs. 8.6 ± 1.8, *P* = 0.02). However, BMI and the serum glucose did not show significant difference. In the subgroup analysis, T2D patients who were on insulin therapy showed a longer disease duration (11.4 ± 7.1 vs. 2.5 ± 3.1, *P* = 0.001) and a higher serum glucose level (185.0 ± 67.2 vs. 129.1 ± 23.2, *P* = 0.021). Age, BMI, and HbA1c level did not show significant difference within subgroups.

**Table 1:** General characteristics of study subjects.

Variable	DM (*n* = 51)	Control (*n* = 51)		T1D			T2D		
				T1D (*n* = 12)	Control (*n* = 12)		T2D (*n* = 39)	Control (*n* = 39)	
	Mean ± SD	Mean ± SD	*P* value	Mean ± SD	Mean ± SD	*P* value	Mean ± SD	Mean ± SD	*P* value
**Age (years)**	55.3 ± 9.0	55.3 ± 9.1	0.987	48.7 ± 12.8	49.1 ± 13.7	0.851	57.3 ± 6.4	57.2 ± 6.2	0.921
**DM duration (years)**	7.8 ± 8.9	–	–	15.9 ± 11.6	–	–	5.2 ± 6.2	–	–
**Male (%)**	60.8	64.7	–	25.0	58.3	–	71.8	66.7	–
BMI (kg/cm^2^)	24.5 ± 2.1	24.5 ± 2.1	0.989	24.0 ± 2.3	23.9 ± 2.4	0.862	24.7 ± 2.1	24.7 ± 2.0	0.948
**Serum glucose (mg/dL)**	151.6 ± 71.6	91.6 ± 10.9	0.000	173.3 ± 122.2	87.2 ± 11.2	0.001	144.9 ± 47.1	93.0 ± 10.5	0.000
**HbA1c (%)**	7.7 ± 1.6	5.8 ± 0.27	0.000	8.6 ± 1.8	5.7 ± 0.26	0.001	7.4 ± 1.4	5.8 ± 0.27	0.001

Data are mean ± standard deviation. DM = diabetes mellitus, T1D = type 1 diabetes, T2D = type 2 diabetes, BMI = body mass index.

### Comparison of the CT texture parameters between the control group and the DM patients

Table 2 shows the comparison of CT texture parameters between DM and control groups. For all patients with diabetes, in the histogram parameters, a diabetic pancreas showed significantly lower mean attenuation, higher standard deviation, higher variance, higher entropy, and higher homogeneity (*P* < .05, respectively). The pancreatic parenchyma of DM patients had a significantly larger surface area and higher sphericity than in the control groups patients (*P* < .05). Regarding the texture parameters, discrete compactness, GLCM contrast, and GLCM entropy were statistically significant (*P* < .05, respectively). A diabetic pancreas had a higher discrete compactness, lower GLCM contrast, and higher GLCM entropy.

**Table 2:** Comparison of CT texture parameters between control group and DM patients.

Variable	DM (*n* = 51)	Control (*n* = 51)		T1D			T2D		
				T1D (*n* = 12)	Control (*n* = 12)		T2D (*n* = 39)	Control (*n* = 39)	
	Mean ± SD	Mean ± SD	*P* value	Mean ± SD	Mean ± SD	*P* value	Mean ± SD	Mean ± SD	*P* value
**Mean attenuation (HU)**	93.2 ± 27.3	110.3 ± 20.4	**0.001**	91.5 ± 24.2	118.0 ± 12.4	**0.004**	93.7 ± 28.4	107.9 ± 21.9	**0.015**
**Standard deviation (HU)**	37.0 ± 10.1	31.8 ± 6.1	**0.002**	33.4 ± 10.8	26.8 ± 4.2	0.060	38.0 ± 9.8	33.3 ± 5.7	**0.013**
**Variance (HU)**	1466.2 ± 862.2	1047.2 ± 414.7	**0.003**^#^	1224.4 ± 901.9	732.8 ± 236.7	0.081	1540.6 ± 847.7	1144.0 ± 411.4	**0.011**
**Skewness**	-1.09 ± 0.38	-1.22 ± 0.68	0.221	-0.95 ± 0.26	-0.81 ± 0.39	0.301	-1.13 ± 0.41	-1.35 ± 0.70	0.113
**Kurtosis**	3.06 ± 2.15	6.32 ± 13.9	0.100	2.09 ± 1.22	3.10 ± 1.34	0.066	3.35 ± 2.30	7.30 ± 15.7	0.133
**Entropy**	4.9 ± 0.27	4.8 ± 0.17	**0.003**	4.8 ± 0.29	4.6 ± 0.13	0.070	4.9 ± 0.26	4.8 ± 0.16	**0.008**
**Homogeneity**	0.017 ± 0.0071	0.013 ± 0.0049	**0.001**	0.017 ± 0.0064	0.010 ± 0.0017	**0.003**	0.017 ± 0.0074	0.013 ± 0.0053	0.191
**Surface Area (mm^2^)**	11205.1 ± 3542.3	9735.1 ± 2125.4	**0.013**	9588.2 ± 4043.6	10180.0 ± 2794.7	0.681	11702.7 ± 3270.7	9598.2 ± 1897.7	**0.001**^#^
**Effective Diameter (mm)**	135.2 ± 29.1	136.6 ± 17.8	0.760	107.3 ± 24.6	139.3 ± 21.2	**0.003**	143.7 ± 24.9	135.8 ± 16.8	0.103
**Volume (cm^3^)**	60.0 ± 24.7	59.6 ± 14.8	0.925	37.9 ± 16.6	62.3 ± 18.2	**0.002**	66.8 ± 22.9	58.8 ± 13.7	0.066
**Sphericity**	0.34 ± 0.037	0.32 ± 0.021	**0.001**^#^	0.35 ± 0.058	0.32 ± 0.021	0.194	0.34 ± 0.029	0.32 ± 0.021	0.769
**Discrete Compactness**	0.13 ± 0.23	-0.0071 ± 0.16	**0.001**	0.080 ± 0.32	0.028 ± 0.17	0.632	0.15 ± 0.19	-0.018 ± 0.15	**0.004**
**GLCM Contrast**	1329.9 ± 559.0	1616.0 ± 590.5	**0.014**^#^	799.7 ± 462.0	1351.9 ± 614.5	**0.021**	1493.1 ± 483.0	1697.3 ± 566.3	0.091
**GLCM Entropy**	4.1 ± 0.20	4.0 ± 0.12	**0.024**^#^	4.0 ± 0.21	3.9 ± 0.11	0.531	4.1 ± 0.19	4.0 ± 0.12	**0.004**
**GLCM ASM**	(1.45 ± 0.67)×10^−4^	(1.63 ± 0.43)×10^−4^	0.106	(1.83 ± 0.80)×10^−4^	(1.94 ± 0.42)×10^−4^	0.710	(1.33 ± 0.59)×10^−4^	(1.54 ± 0.39)×10^−4^	0.157
**GLCM IDM**	0.056 ± 0.018	0.055 ± 0.0074	0.899	0.070 ± 0.020	0.059 ± 0.008	0.090	0.051 ± 0.014	0.054 ± 0.007	0.356
**GLCM Moments**	1.2 ± 0.36	1.4 ± 0.32	0.072	1.1 ± 0.39	1.4 ± 0.28	**0.038**	1.3 ± 0.34	1.3 ± 0.33	0.804

DM = diabetes mellitus, T1D = type 1 diabetes, T2D = type 2 diabetes, SD = standard deviation, BMI = body mass index, GLCM = gray level co-occurrence matrices, ASM = angular second moment, IDM = inverse difference moment.

* Independent sample t test with its corresponding control group.

# Significant variables on multivariable analysis

For patients with T1D, several texture parameters showed a significant difference between the T1D and the control groups. T1D pancreas showed a lower mean attenuation, greater homogeneity, smaller effective diameter, smaller volume, lower GLCM contrast, and lower GLCM moments (*P* < .05). For patients with T2D, T2D group showed a lower mean attenuation, higher standard deviation, higher variance, higher entropy, larger surface area, higher discrete compactness, and higher GLCM entropy (*P* < .05). The summary statistics of the extracted CT features in the T2D and its subgroups are in the S2 Appendix. T2D patients who were not receiving insulin therapy showed a lower mean attenuation, higher standard deviation, higher variance, higher entropy, higher homogeneity, a larger surface area, higher sphericity, higher discrete compactness, higher GLCM entropy, lower GLCM ASM, and lower GLCM IDM (*P* < .05). Logistic regression analysis revealed that a higher variance (adjusted odds ratio, 1.003; *P* = .016) and sphericity (adjusted odds ratio, 2.095×10^13^; *P* = .045) were statistically significant independent differentiators of T2D patients without insulin therapy. On the other hand, for the insulin-treated T2D group, no texture parameter showed a significant difference on logistic regression analysis.

### Important CT texture parameters for predicting diabetes

**Fig 3:**
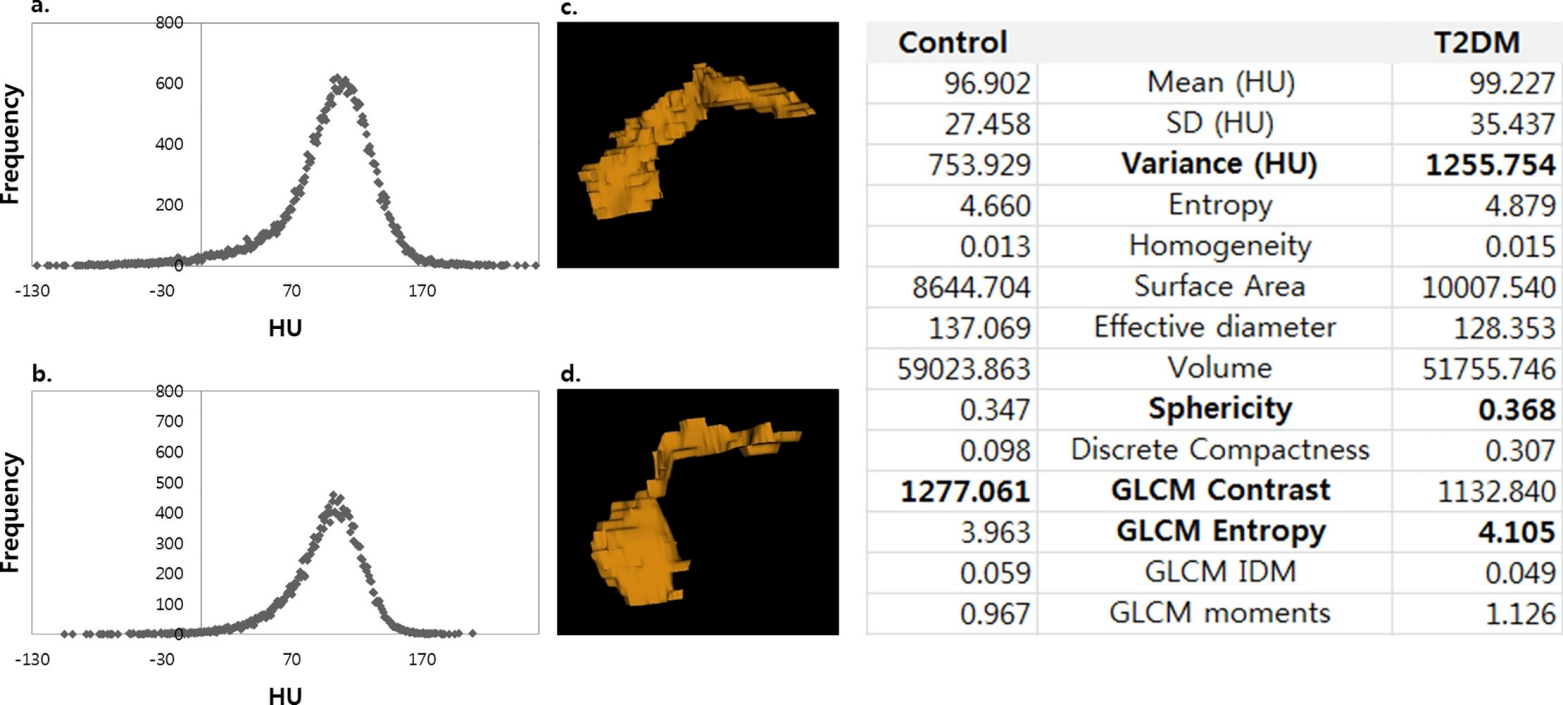
3D reconstruction images of an (a) T2D pancreas and (b) its control, with their histograms representing texture features. Each image is from 58-year-old female, with BMI of 24.6 and 24.2, respectively. The T2D patient was not on non-insulin therapy. (c, d) Texture parameters of T2D patients show consistent results with multivariate analysis, including a higher variance (1255.754 HU vs. 753.929 HU), higher sphericity (0.368 vs. 0.347), higher GLCM entropy (4.105 vs. 3.963), and lower GLCM contrast (1132.840 vs. 1277.061).

**Fig 4:**
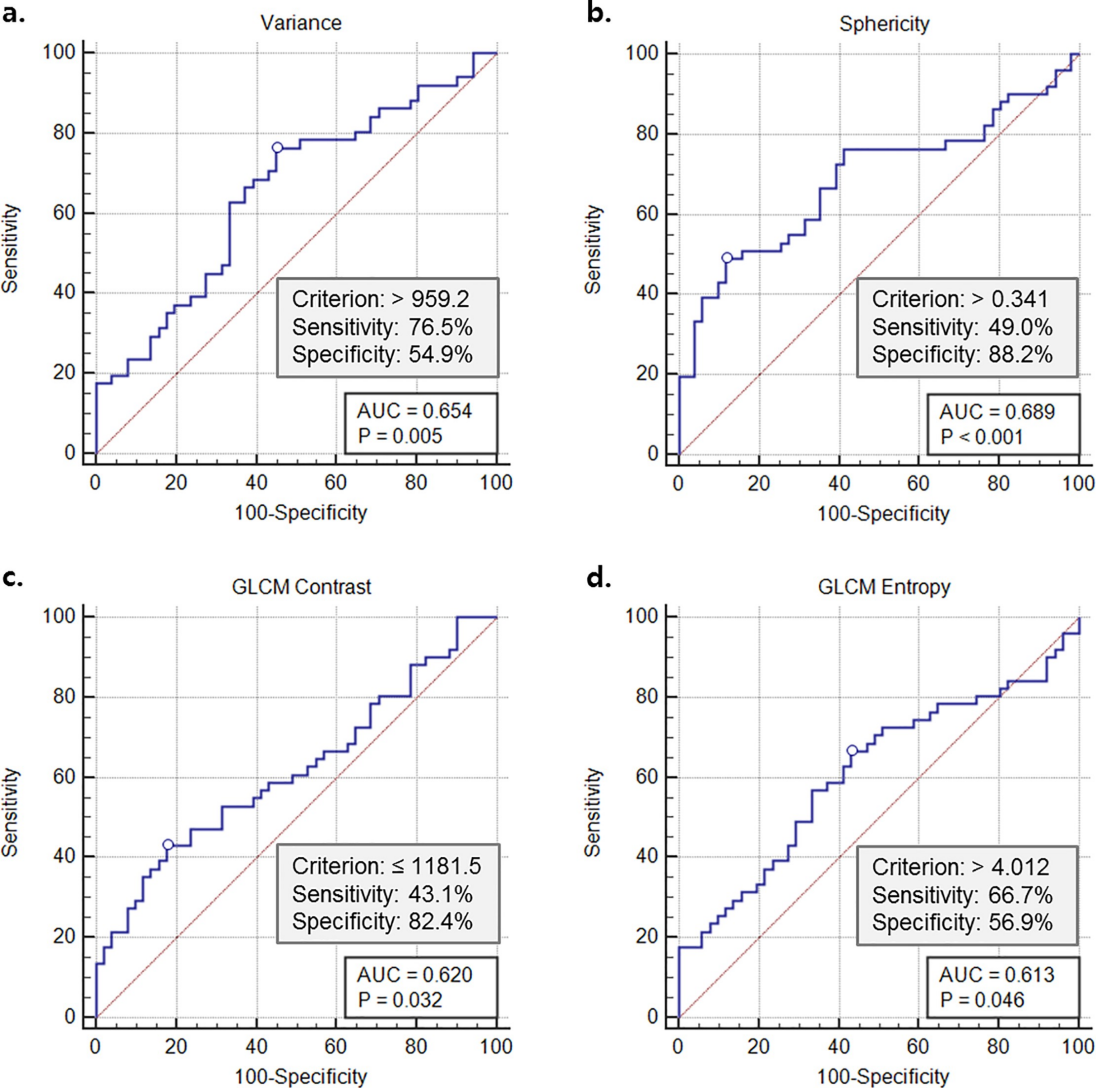
Receiver operating characteristic (ROC) curve for (a) variance, (b) sphericity, (c) GLCM contrast, and (d) GLCM entropy for differentiation between diabetes and normal control.

A higher variance (1466.2 ± 862.2 vs. 1047.2 ± 414.7, adjusted odds ratio, 1.002; *P* = .005), sphericity (0.34 ± 0.037 vs. 0.32 ± 0.021, adjusted odds ratio, 1.649×10^4^; *P* = .048), GLCM entropy (4.1 ± 0.20 vs. 4.0 ± 0.12, adjusted odds ratio, 1.057×10^5^; *P* = .032), and lower GLCM contrast (1329.9 ± 559.0 vs. 1616.0 ± 590.5, adjusted odds ratio, 0.997; *P* < .001) were statistically significant independent differentiators of a diabetic pancreas from a normal pancreas (Table 3, Fig 3). When we constructed ROC curves, the optimal threshold value for variance was 959.2 with 76.5% sensitivity and 54.9% specificity, and the optimal threshold value for sphericity was 0.341 with 49.0% sensitivity and 88.2% specificity. For GLCM contrast, the optimal threshold value was 1181.5 with 43.1% sensitivity and 82.4% specificity. GLCM entropy had an optimal threshold value of 4.012 with 66.7% sensitivity and 56.9% specificity. The area under the curve (AUC) ranged from 0.613 to 0.689 (Fig 4). In a T2D pancreas, the larger surface area was the only variable that was significant (adjusted odds ratio, 1000.382×10^−3^; *P* = 0.025, Table 3). Features including skewness, kurtosis, an effective diameter, volume, GLCM contrast, GLCM entropy, and GLCM IDM showed significant differences between T1D and T2D patients (*P* < .05) (S3 Appendix).

**Table 3:** Logistic regression analysis for distinguishing DM patients from control group.

	Control vs. DM (*n* = 51)				Control vs. T1D (*n* = 12)		Control vs. T2D (*n* = 39)			
Variable	Univariable		Multivariable		Univariable		Univariable		Multivariable	
	OR (95% CI)	*P* value	OR (95% CI)	*P* value	OR (95% CI)	*P* value	OR (95% CI)	*P* value	OR (95% CI)	*P* value
**Mean attenuation (HU)**	0.978 (0.935–1.022)	0.316			0.050 (0.000-)	0.994	1.006 (0.972–1.042)	0.720		
**Standard deviation (HU)**	0.838 (0.444–1.579)	0.584					0.590 (0.229–1.521)	0.275		
**Variance (HU)**	1.004 (0.996–1.012)	0.369	1.002 (1.001–1.004)	**0.005**			1.006 (0.994–1.018)	0.321		
**Entropy**	6.477 (0.263–159.8)	0.253					16.361 (0.047–5756.49)	0.350		
**Homogeneity**	0.103×10^−133^ (0.204×10^−270^–524.3)	0.055								
**Surface Area (mm^2^) ×10^3^**	1000.006 (999.768–1000.244)	0.960					1000.382 (999.987–1000.777)	0.058	1000.252 (1000.032–1000.472)	**0.025**
**Effective diameter (mm)**					0.001 (0.000-)	0.998				
**Volume (cm^3^)**					1.007 (0.153×10^−3^–6.620×10^3^)	0.999	1.000 (1.000–1.000)	0.127		
**Sphericity ×10^−4^**	4.017×10^2^ (9.868×10^−4^–1.635×10^8^)	**0.021**	1.649 (1.099×10^−4^–2.472×10^4^)	**0.048**						
**Discrete Compactness**	0.185 (0.005–7.179)	0.366					18.345 (0.186–1811.545)	0.214		
**GLCM Contrast**	0.997 (0.995–0.999)	**0.001**	0.997 (0.995–0.998)	**0.000**	0.919 (0.400×10^−11^–2.109×10^11^)	0.995				
**GLCM Entropy ×10^−5^**	5.284 (6.448×10^−5^–4.331×10^5^)	**0.022**	1.057 (2.683×10^−5^–4.164×10^5^)	**0.032**			4.258×10^−3^ (1.143×10^−6^–15.859)	0.149		

DM = diabetes mellitus, T1D = type 1 diabetes, T2D = type 2 diabetes, CI = confidence interval, BMI = body mass index, GLCM = gray level co-occurrence matrices.

## Discussion

Our study showed that higher variance (adjusted OR, 1.002; *P* = .005), sphericity (adjusted OR, 1.649×10^4^; *P* = .048), GLCM entropy (adjusted OR, 1.057×10^5^; *P* = .032), and lower GLCM contrast (adjusted OR, 0.997; *P* < .001) were statistically significant differentiators of diabetic pancreas from normal pancreas. In the subgroup analysis, only a larger surface area (adjusted OR, 1.000; *P* = .025) was a significant predictor for T2D.

Early detection of diabetes will help to prevent or delay the vascular complications and therefore reduce the clinical, social, and economic burden of the disease. As CT is widely used imaging modality, it would be useful if we could perform an additional texture analysis of the pancreas in order to extract important features that indicate diabetes. Indeed, the recent advancements of imaging studies of the pancreas provide new information regarding the pathophysiology of diabetes. For example, previous studies had shown that the pancreatic volume was lower in T1D or T2D patients compared with that in normal patients [6, 7]. Other studies attempted to analyze the association between pancreatic adipose tissue infiltration and endocrine function using the CT attenuation difference [16] or MR spectroscopy [17]. However, to our knowledge there are no studies that attempted to quantitatively assess diabetic pancreas by using the texture analysis. Imaging texture analysis is an emerging area of “radiomics” that extracts, analyzes, and interprets quantitative imaging features [18]. Texture analysis allows objective assessment of lesion and organ heterogeneity beyond what is possible with subjective visual interpretation and thus may reflect information regarding the tissue microenvironment.

Sphericity is a term that indicates a measure of how round an object is. The value of sphericity is defined as the ratio of the volume of a nodule and the volume of a minimum, circumscribed sphere. The index of sphericity considers two parameters, i.e. the shape perimeter measured on a 2D surface and a relative parameter, namely the mean shape diameter. The mean shape diameter is determined from the perimeter and the area of the irregular shape [19]. There are previous studies which reported that pancreatic lobulation is increased in diabetic patients [20, 21], and which corresponds to our result of an increased surface area in a diabetic pancreas. However, as insulin acts as a trophic factor on the exocrine pancreas, the pancreatic mass shrinks with the natural course of diabetes. This histologic change may lead to an overall increased sphericity of the pancreas in diabetic patients.

GLCM entropy indicates randomness of the matrix, and thus reflecting tissue heterogeneity [22]. For example, malignant lymph nodes showed higher GLCM entropy in the previous study assessing mediastinal nodes in lung cancer [23]. A heterogeneous image has a high value regarding the GLCM entropy [24], and therefore our results suggest that the tissue heterogeneity of the pancreas in diabetic patients is higher. As only a few studies have investigated the parenchymal texture of the pancreas, there is a relative paucity of data describing the association between the texture parameter and histological changes. Increased inflammation and fibrosis in a diabetic pancreas may result in an altered distribution and composition of pancreatic cells, and thus resulting in increased heterogeneity. This is an area which needs to be verified by further studies.

GLCM contrast measures the local variations present in an image. Variance, on the other hand, is calculated from the original image values and does not consider pixel neighborhood relationships [25]. These two, similar but different parameters showed the opposite result in pancreatic texture analysis and with lower GLCM contrast and higher variance in diabetic patients. The basic difference is that first-order statistics estimate the properties of individual pixel values, while ignoring the spatial interaction between image pixels, whereas second-order statistics estimate the properties of two or more pixel values occurring at specific locations relative to each other [26]. When we apply our results to histopathologic changes, we may infer that a diabetic pancreas exhibits overall increased variance, while the neighboring cell structures become rather similar to each other. In the presence of diabetes, not only inflammatory changes in the exocrine pancreas, but also other pathologic alterations, such as atrophy, fibrosis, and fat deposition, occur [27]. To reveal the radiologic-pathologic correlation regarding textural features and pathologic correlates, further studies are warranted.

Apart from the intrinsic limits of any retrospective study, our study has several limitations. First, although we have selected two CT scanners, our examination protocol has some variances among patients. This may have caused the effect of CT attenuation values on each voxel. Second, manual segmentation was applied to assign a region of interest, which can be labor-intensive and limited by inter- and intra-observer reproducibility. Third, factors regarding the severity and duration of diabetes were not counted during the analysis. Recent studies have shown that a diabetic pancreas is more prone to inflammatory changes and fibrosis [28], which is likely to result in textural alteration over time. Although we matched normal patients in order to minimize the effect of age and BMI differences, the variable duration and severity of diabetes between the subgroups could have functioned as confounding factors. Finally, relatively few T1D patients were included in this study. Unlike T2D which is caused by insulin resistance, T1D is an autoimmune disorder resulting in the destruction of the pancreatic ß-cells that secrete insulin [29]. Although the endocrine compartment constitutes only 1–3% of the entire pancreas volume [19, 30], this pathophysiological difference caused different histological changes in the two patient groups. Indeed, our study showed greater volume reduction in T1D patients than that observed in patients with T2D, which corresponds well with the results of previous studies [7, 9, 31]. However, no texture parameters remained significant after multivariable analysis in the T1D group. A further study that includes a larger patient population is warranted in order to determine the textural characteristics of a T1D pancreas.

In conclusion, computerized CT texture analysis of the pancreas allows objective assessment of its parenchyma, which can be useful in differentiating diabetes from normal. This study reveals that higher variance, sphericity, GLCM entropy, and lower GLCM contrast are significant differentiators between DM and normal patients in 3D CT texture analysis of the pancreas.

## Supporting information

S1 Appendix The detailed information regarding the texture features. (DOCX)

S2 Appendix Comparison of CT texture parameters between control group and T2DM patients, depending on insulin use. (DOCX)

S3 Appendix Comparison of CT texture parameters between control group and DM patients. (DOCX)
