# Supplementary material for: Application of computerized 3D-CT texture analysis of pancreas for the assessment of patients with diabetes
Source: PLoS One. 2020 Jan 13;15(1):e0227492. doi: 10.1371/journal.pone.0227492 (PMC6957148; doi:10.1371/journal.pone.0227492)
Supplement: S1 Appendix — (DOCX) [file pone.0227492.s001.docx]

**S1 Appendix. The detailed information regarding the texture features.**

**Discrete Compactness**

The discrete compactness of an object is calculated as the ratio between the contact surface area (Ac) and the theoretical maximum contact surface area (Acmax). The contact surface area is defined as the sum of the surface areas where two adjacent voxels are in common contact. The value of discrete compactness varies from 0 to 1, and the value is related with the circularity of the object. The discrete compactness is calculated by the following equation. The Acmin is minimum contact surface area.


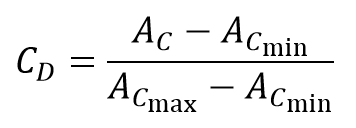


**Texture Features from Gray- level Co-occurrence Matrix**

A gray-level co-occurrence matrix (GLCM) is composed of the element P (i, j) which corresponds relative frequency with co-occurrence of the pair of pixels with grey level intensity i and j, separated by a given distance and a given direction. Various texture parameters derived from the GLCM matrix can be used in image analysis. The five different texture parameters derived from GLCM matrix were used in this study [1].

GLCM moments is can be defined as following equation.


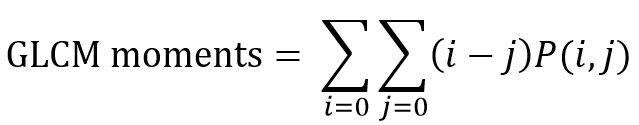


GLCM angular second moment reflects the homogeneity of a given image. The more homogeneous image represents higher GLCM angular second moment.


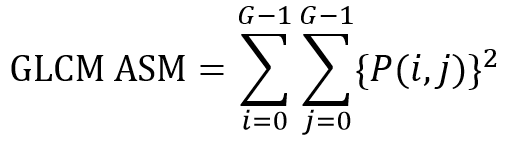


GLCM inverse difference moment is also a measure of the homogeneity of the image. The homogeneous image gives relatively higher inverse difference moment.


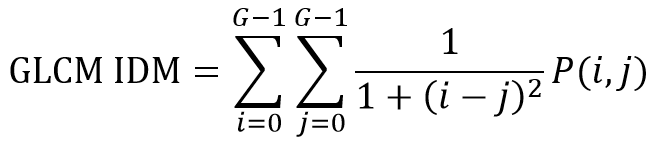


GLCM contrast is a measure of local gray level intensity variation.


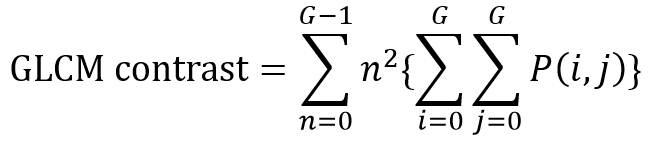

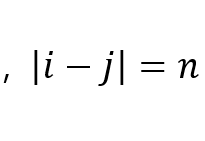


GLCM entropy reflects homogeneity of image. Homogeneous image has high value of the GLCM entropy.


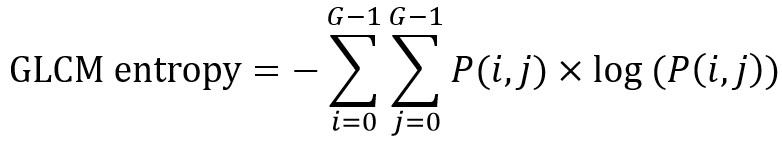


**References**

1. Albregtsen F. Statistical texture measures computed from gray level coocurrence matrices. Image processing laboratory, department of informatics, university of oslo. 2008;5.
