# Supplementary material for: Application of computerized 3D-CT texture analysis of pancreas for the assessment of patients with diabetes
Source: PLoS One. 2020 Jan 13;15(1):e0227492. doi: 10.1371/journal.pone.0227492 (PMC6957148; doi:10.1371/journal.pone.0227492)
Supplement: S2 Appendix — (DOCX) [file pone.0227492.s002.docx]

**S2 Appendix.** **Comparison of CT texture parameters between control group and T2DM patients, depending on insulin use**

| Variable | Control (*n* = 39) | T2DM (*n* = 39) | | T2DM  not on insulin (*n* = 28) | | T2DM  on insulin (*n* = 11) | |
| --- | --- | --- | --- | --- | --- | --- | --- |
|  | Mean ± SD | Mean ± SD | *P* value | Mean ± SD | *P* value^*^ | Mean ± SD | *P* value^*^ |
| Mean attenuation (HU) | 107.9 ± 21.9 | 93.7 ± 28.4 | **0.015** | 98.7 ± 29.3 | **0.048** | 81.2 ± 22.8 | 0.127 |
| Standard deviation (HU) | 33.3 ± 5.7 | 38.0 ± 9.8 | **0.013** | 37.5 ± 9.2 | **0.004** | 39.5 ± 11.5 | 0.662 |
| Variance (HU) | 1144.0 ± 411.4 | 1540.6 ± 847.7 | **0.011** | 1486.8 ± 771.1 | **0.005**^#^ | 1677.8 ± 1047.2 | 0.533 |
| Skewness | -1.35 ± 0.70 | -1.13 ± 0.41 | 0.113 | -1.13 ± 0.38 | 0.120 | -1.14 ± 0.49 | 0.572 |
| Kurtosis | 7.30 ± 15.74 | 3.35 ± 2.30 | 0.133 | 3.50 ± 2.38 | 0.134 | 3.01 ± 2.15 | 0.802 |
| Entropy | 4.80 ± 0.16 | 4.92 ± 0.26 | **0.008** | 4.91 ± 0.24 | **0.004** | 4.94 ± 0.31 | 0.717 |
| Homogeneity | 0.013 ± 0.0053 | 0.017 ± 0.0074 | 0.191 | 0.016 ± 0.0073 | **0.041** | 0.020 ± 0.007 | 0.201 |
| Surface Area (mm^2^) | 9598.2 ± 1897.7 | 11702.7 ± 3270.7 | **0.001**^#^ | 11254.7 ± 2954.6 | **0.005** | 12842.9 ± 3884.2 | 0.069 |
| Effective Diameter (mm) | 135.8 ± 16.8 | 143.7 ± 24.9 | 0.103 | 143.6 ± 24.3 | 0.122 | 144.2 ± 27.5 | 0.575 |
| Volume (cm^3^) | 58.8 ± 13.7 | 66.8 ± 22.9 | 0.066 | 66.5 ± 22.2 | 0.091 | 67.5 ± 25.7 | 0.470 |
| Sphericity | 0.32 ± 0.021 | 0.34 ± 0.029 | 0.769 | 0.33 ± 0.027 | **0.028**^#^ | 0.35 ± 0.031 | **0.018** |
| Discrete Compactness | -0.018 ± 0.15 | 0.15 ± 0.19 | **0.004** | 0.10 ± 0.18 | **0.004** | 0.26 ± 0.19 | **0.004** |
| GLCM Contrast | 1697.3 ± 566.3 | 1493.1 ± 483.0 | 0.091 | 1589.0 ± 396.3 | 0.647 | 1248.9 ± 609.0 | **0.032** |
| GLCM Entropy | 4.04 ± 0.12 | 4.13 ± 0.19 | **0.004** | 4.13 ± 0.17 | **0.003** | 4.13 ± 0.25 | 0.907 |
| GLCM ASM | (1.54 ± 0.39)×10^-4^ | (1.33 ± 0.59)×10^-4^ | 0.157 | (1.27 ± 0.49)×10^-4^ | **0.005** | (1.46 ± 0.80)×10^-4^ | 0.634 |
| GLCM IDM | 0.054 ± 0.007 | 0.051 ± 0.014 | 0.356 | 0.048 ± 0.008 | **0.001** | 0.059 ± 0.023 | 0.303 |
| GLCM Moments | 1.34 ± 0.33 | 1.27 ± 0.34 | 0.804 | 1.35 ± 0.32 | 0.753 | 1.09 ± 0.33 | 0.248 |

Note.━ GLCM = gray level co-occurrence matrices, ASM=angular second moment, IDM = inverse difference moment

* Independent sample *t* test with its corresponding control group. # Significant variables on multivariable analysis
