# Supplementary material for: Application of computerized 3D-CT texture analysis of pancreas for the assessment of patients with diabetes
Source: PLoS One. 2020 Jan 13;15(1):e0227492. doi: 10.1371/journal.pone.0227492 (PMC6957148; doi:10.1371/journal.pone.0227492)
Supplement: S3 Appendix — (DOCX) [file pone.0227492.s003.docx]

**S3 Appendix. Comparison of CT texture parameters between control group and DM patients**

| Variable | DM (*n* = 51) | | T1DM (*n* = 12) | | T2DM (*n* = 39) | | T1 vs. T2 |
| --- | --- | --- | --- | --- | --- | --- | --- |
|  | Mean ± SD | *P* value | Mean ± SD | *P* value^*^ | Mean ± SD | *P* value^*^ | *P* value^$^ |
| Mean attenuation (HU) | 93.2 ± 27.3 | **0.001** | 91.5 ± 24.2 | **0.004** | 93.7 ± 28.4 | **0.015** | 0.564 |
| Standard deviation (HU) | 37.0 ± 10.1 | **0.002** | 33.4 ± 10.8 | 0.060 | 38.0 ± 9.8 | **0.013** | 0.176 |
| Variance (HU) | 1466.2 ± 862.2 | **0.003**^#^ | 1224.4 ± 901.9 | 0.081 | 1540.6 ± 847.7 | **0.011** | 0.176 |
| Skewness | -1.09 ± 0.38 | 0.221 | -0.95 ± 0.26 | 0.301 | -1.13 ± 0.41 | 0.113 | **0.048** |
| Kurtosis | 3.06 ± 2.15 | 0.100 | 2.09 ± 1.22 | 0.066 | 3.35 ± 2.30 | 0.133 | **0.035** |
| Entropy | 4.9 ± 0.27 | **0.003** | 4.8 ± 0.29 | 0.070 | 4.9 ± 0.26 | **0.008** | 0.230 |
| Homogeneity | 0.017 ± 0.0071 | **0.001** | 0.017 ± 0.0064 | **0.003** | 0.017 ± 0.0074 | 0.191 | 0.842 |
| Surface Area (mm^2^) | 11205.1 ± 3542.3 | **0.013** | 9588.2 ± 4043.6 | 0.681 | 11702.7 ± 3270.7 | **0.001**^#^ | 0.083 |
| Effective Diameter (mm) | 135.2 ± 29.1 | 0.760 | 107.3 ± 24.6 | **0.003** | 143.7 ± 24.9 | 0.103 | **0.000** |
| Volume (cm^3^) | 60.0 ± 24.7 | 0.925 | 37.9 ± 16.6 | **0.002** | 66.8 ± 22.9 | 0.066 | **0.000** |
| Sphericity | 0.34 ± 0.037 | **0.001**^#^ | 0.35 ± 0.058 | 0.194 | 0.34 ± 0.029 | 0.769 | 0.912 |
| Discrete Compactness | 0.13 ± 0.23 | **0.001** | 0.080 ± 0.32 | 0.632 | 0.15 ± 0.19 | **0.004** | 0.450 |
| GLCM Contrast | 1329.9 ± 559.0 | **0.014**^#^ | 799.7 ± 462.0 | **0.021** | 1493.1 ± 483.0 | 0.091 | **0.000** |
| GLCM Entropy | 4.1 ± 0.20 | **0.024**^#^ | 4.0 ± 0.21 | 0.531 | 4.1 ± 0.19 | **0.004** | **0.020** |
| GLCM ASM | (1.45 ± 0.67)×10^-4^ | 0.106 | (1.83 ± 0.80)×10^-4^ | 0.710 | (1.33 ± 0.59)×10^-4^ | 0.157 | 0.069 |
| GLCM IDM | 0.056 ± 0.018 | 0.899 | 0.070 ± 0.020 | 0.090 | 0.051 ± 0.014 | 0.356 | **0.003** |
| GLCM Moments | 1.2 ± 0.36 | 0.072 | 1.1 ± 0.39 | **0.038** | 1.3 ± 0.34 | 0.804 | 0.143 |

Note.━ * Independent sample t test with its corresponding control group. # Significant variables on multivariable analysis

$ Mann Whitney U-test between T1DM and T2DM groups.
